# Ultrasonography-guided drainage versus surgical drainage for deep neck space abscesses: a systematic review and meta-analysis

**DOI:** 10.1017/S0022215124000501

**Published:** 2024-09

**Authors:** Mohammad Alzaid, Mohammed Ramadhan, Ahmad Abul, Mohammad Karam, Abdulmalik Alsaif, Emma Stapleton

**Affiliations:** 1 School of Medical Sciences, University of Manchester, Manchester, UK; 2 Department of Surgery, Jaber Al-Ahmed Hospital, Kuwait City, Kuwait; 3 Division of Surgical and Interventional Sciences, University College London, London, UK; 4 Department of Ophthalmology & Visual Sciences, McGill University, Montreal, Canada; 5 Royal Eye Unit, Kingston Hospital Foundation Trust, London, UK; 6 Department of Otolaryngology, Manchester Royal Infirmary, Manchester, UK

**Keywords:** ultrasonography, abscess, retropharyngeal abscess, drainage, length of stay, cost savings

## Abstract

**Objective:**

To compare ultrasonography-guided drainage versus conventional surgical incision and drainage in deep neck space abscesses.

**Methods:**

The study was pre-registered on the National Institute of Health Research Prospective Register of Systematic Reviews (CRD42023466809) and adhered to Preferred Reporting Items for Systematic Reviews and Meta-Analyses guidelines. The Medline, Embase and Central databases were searched. Primary outcomes were length of hospital stay and recurrence. Heterogeneity and bias risk were assessed, and a fixed-effects model was applied.

**Results:**

Of 646 screened articles, 7 studies enrolling 384 participants were included. Ultrasonography-guided drainage was associated with a significantly shorter hospital stay (mean difference = −2.31, *p* < 0.00001), but no statistically significant difference was noted in recurrence rate compared to incision and drainage (odds ratio = 2.02, *p* = 0.21). Ultrasonography-guided drainage appeared to be associated with cost savings and better cosmetic outcomes.

**Conclusion:**

Ultrasonography-guided drainage was associated with a shorter hospital stay, making it a viable and perhaps more cost-effective alternative. More randomised trials with adequate outcomes reporting are recommended to optimise the available evidence.

## Introduction

The neck is a complex structure, with superficial and multilayered deep fascia forming several potential spaces among the fascial planes. Deep neck space abscesses can develop from infectious involvement of these spaces and planes, most commonly following dental and pharyngotonsillitis infections.^1,2^ Submandibular space is most implicated (around 42.3 per cent of cases), followed by paraphyngeal and parotid spaces at 21.15 and 11.53 per cent, respectively.^3^ The primary complaint varies depending on the involved space and can include fever, pain, swelling, trismus, dysphagia and odynophagia.^4^

Improper control of infection can result in significant complications such as descending necrotising mediastinitis, pneumonia, jugular vein thrombosis, carotid artery erosion and septic shock, with a mortality rate of up to 50 per cent.^5–7^ The incidence of deep neck space abscesses had decreased with antibiotics and improved dental hygiene, but this trend has been reversed over the past 10 years.^8^

Traditionally, surgical incision and drainage performed intra-orally or extra-orally, coupled with antibiotics coverage, has been the mainstay treatment for deep neck space abscesses.^9,10^ Several drawbacks remained unaddressed despite the proven efficacy of incision and drainage in the literature. Patients often undergo general anaesthesia and require an airway secured with tracheostomy or fibre-optic nasal intubation. In addition, the intra-oral approach could be complicated by purulent discharge or persistent bleeding, worsening already limited visualisation and, on some occasions, leading to airway compromise. When performed extra-orally, the surgeon often requires neck incision and exploration, which carries the inherent risk of neurovascular injury on top of the cosmetically undesirable scar. Rarely, tumour dissemination could occur following incision and drainage in patients with deep neck space infection caused by malignancy.^11,12^

More recently, several studies have advocated that ultrasonography-guided drainage is minimally invasive and an effective alternative to incision and drainage, obviating the abovementioned drawbacks.^10,11,13–16^ With this readily available and inexpensive tool, surgeons can insert the puncture tube under real-time imaging guidance. This is particularly important as abscess development is dynamic, requiring accurate puncture timing and subsequent drainage monitoring.^11^

To our knowledge, this is the first systematic review and meta-analysis to compare ultrasonography-guided drainage to incision and drainage in adult patients with deep neck space abscesses, focusing on the length of hospital stay and recurrences as primary outcomes. The reported complications, cosmetic appearance and/or scar formation, and cost savings were also studied.

## Materials and methods

### Registration

This systematic review and meta-analysis adhered to the Preferred Reporting Items for Systematic Reviews and Meta-Analyses 2020 statement and the instructions published by Sataloff *et al*.^17,18^ The study protocol was registered a priori with the National Institute of Health Research Prospective Register of Systematic Reviews, registration number CRD42023466809.

### Data sources and literature search

A comprehensive systematic search of the Medline, Embase and Central databases was performed in October 2023 without any language or geographical restrictions. A combination of free text, medical subject headings terms and Boolean logical operators was used to construct the search strategy after consultation with a literature search expert. The reference list of the included studies and ‘cited by’ articles was also screened for relevance. Key ENT journals were manually searched, including *JAMA Otolaryngology – Head & Neck Surgery*, *European Archives of Oto-Rhino-Laryngology* and *The Journal of Laryngology and Otology*. Specific databases (OpenMD, MedNar and BASE) specialising in grey literature were briefly searched. A search was conducted using the following keywords: deep neck space OR deep neck abscess OR deep neck abscesses OR deep neck infections OR deep neck infection OR neck abscess OR neck infections OR DNSIs OR parapharyngeal OR retropharyngeal OR submandibular, AND ultrasound OR ultrasonography OR ultrasound-guided OR ultrasonography-guided, AND incision OR surgical.

### Eligibility criteria

To identify the totality of relevant literature, all randomised control trials and observational studies on deep neck space abscesses comparing ultrasonography-guided drainage with incision and drainage that reported at least one clinical outcome of interest were deemed eligible for inclusion. The interventional group of interest was draining with ultrasound, and the comparator was surgical incision. Participants were adults (aged 16 years and above) with a clinical diagnosis of deep neck space abscess. No gender, ethnicity or morbidity status restrictions were applied. Duplicates, case reports, case series, review articles, conference abstracts, opinion pieces, single-arm observational studies and studies in non-English languages without translation were excluded. Paediatric patients under the age of 16 years were excluded.

### Outcomes

The primary outcomes were length of hospital stay and recurrence. The secondary outcomes included scar formation and/or cosmetic appearance, reported complications and cost savings.

### Process of screening and data extraction

Two reviewers (MA and MR) independently screened titles and abstracts. Once shortlisted, full texts of all potentially eligible papers were retrieved and assessed for our inclusion criteria. Discrepancies in study selection were resolved by consulting the senior author (ES), who provided an unbiased expert perspective for the final determination of the inclusion and/or exclusion of the article.

A standardised Excel spreadsheet was created in keeping with Cochrane's data collection form for intervention reviews. A spreadsheet pilot test was performed, extracting data from random articles and adapting it where necessary. Two independent authors (MA and MR) conducted data extraction. An attempt was made to contact the corresponding authors of relevant studies to share study-level anonymised data regarding missing data, particularly the standard deviation for our outcomes of interest. However, weeks after the first attempt at contact, no replies had been received. The extracted data included first author, publication year, study design, participant demographics (gender, age and co-morbidity where reported), length of follow up and outcomes of interest as above.

### Risk of bias and quality assessment

Two independent authors (AA and AA) assessed the quality of the included studies, and any discrepancies were resolved by consulting ES. For the observational studies, the risk of bias was assessed using the Risk Of Bias In Non-randomised Studies – of Interventions scale, endorsed by the Cochrane organisation.^19^ This tool covers seven domains with ‘signalling questions’ to facilitate judgements regarding the risk of bias, and the judgements of each domain are carried forward to calculate an overall bias risk score. The assessed domains include (1) bias due to confounding, (2) bias in the selection of participants into the study, (3) bias in the classification of interventions, (4) bias due to deviations from intended interventions, (5) bias due to missing data, (6) bias in the measurement of outcomes and (7) bias in the selection of the reported result.

The quality of randomised controlled trials (RCTs) was evaluated using the Cochrane risk-of-bias tool version 2.0 for randomised trials, which comprises five distinct domains from which risk of bias can be ascertained to produce an overall bias score.^20^ These domains include (1) bias arising from the randomisation process, (2) bias due to deviations from intended intervention, (3) bias due to missing outcome data, (4) bias in the measurement of the outcome and (5) bias in the selection of the reported result.

### Statistical analysis

Statistical analysis was conducted using Review Manager version 5.4 and Microsoft Excel. The means difference was measured and the dichotomous outcomes were assessed with an odds ratio for continuous variables such as the length of hospital stay. Heterogeneity was assessed using Cochrane's *Q*-test (*χ*^2^) and inconsistency was quantified by calculating *I*^2^. The heterogeneity was interpreted as 0–25 per cent (low heterogeneity), 25–75 per cent (moderate heterogeneity) and 75–100 per cent (high heterogeneity). Because of the low heterogeneity in this study, a fixed-effect model was used. Reported outcomes were represented in the forest plot at 95 per cent confidence intervals (CIs). A value of *p* less than 0.05 was considered statistically significant.

## Results and analysis

### Literature search results

**Figure 1.**
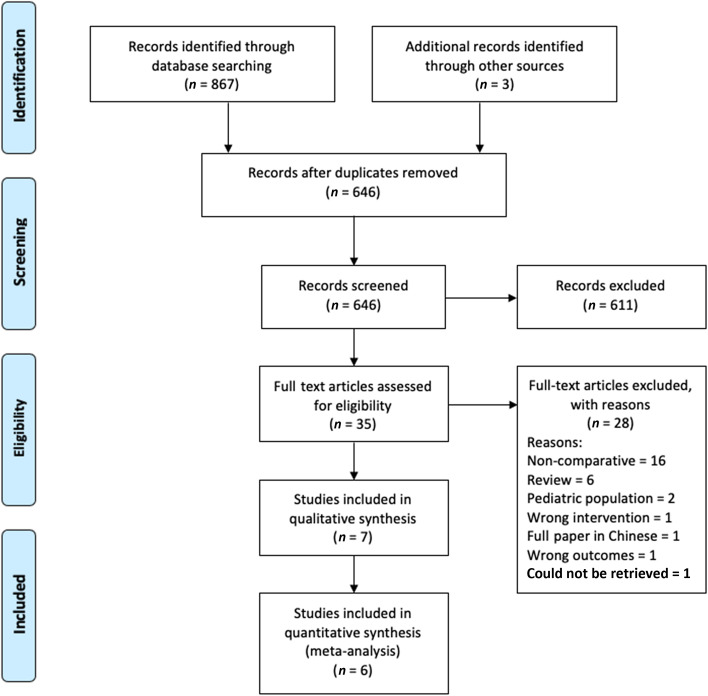
Prisma flow diagram. The Preferred Reporting Items for Systematic Reviews and Meta-Analyses diagram details the search and selection processes applied during the overview.

The last search was conducted on 13 November 2023. The search strategy retrieved 864 studies, and hand-searching and/or snowballing of references and articles ‘cited by’ identified three additional papers. After thoroughly screening the retrieved articles, the authors identified seven studies that met the eligibility criteria (Figure 1).

### Description of studies

Table 1 summarises the included studies' baseline characteristics, with a total sample size of 384 participants. Table 1/2 lists the lesion locations in each study. The studies were standardised in population and design, comparing ultrasonography-guided drainage with incision and drainage for deep neck space abscessess.^21–27^

**Table 1.** Baseline characteristics of the included studies

Study (year)	Journal, country	Study design	Age (USD:I&D (range); years)	Sex (M:F)	Sample (USD:I&D (range); *n*)	Mean abscess volume (USD:I&D; ml)
Biron *et al*. (2013)	*Journal of Otolaryngology – Head and Neck Surgery*, Canada	RCT – sealed envelopes. Not blinded.	31.2:44.3	≈1:1	8:9 (17)	21:14.7 (*p* = 0.25)
Hassan and Gaafar (2021)	Not journal published Poster at ALEXMED ePosters, University of Alexandria, Egypt	RCT, block randomisation	NA	NA	16:16 (32)	NA
Fan and Tao (2021)	*Gland Surgery,* China	Observational study	55.8 (28–76):52.3 (27–75)	11:37	43:17 (60)	8.7:8.8 (*p* = 0.97)
Dabirmoghaddam *et al*. (2017)	*The Journal of Laryngology & Otology*, Iran	Comparative case–control study with sealed envelope randomisation	34.97:35.73	25:33	30:30 (60)	13.03:NA
Limardo *et al*. (2022)	*Acta otorrinolaringológica española*, Argentina	RCT	Overall: 27.3 (15–62)	77:51	64:64 (128)	NA
Strassen *et al*. (2022)	*Journal of Clinical Medicine*, Germany	Retrospective observational study	51.78 (17–81):58.68 (22–90)	25:33	18:39 (57)	5.7:10.1 (*p* = 0.244)
Mallick *et al*. (2023)	*Journal of Cardiovascular Disease Research*, India	Retrospective observational study	49.58 (24–66):58.55 (46–70)	11:19	12:18 (30)	NA

USD = ultrasonography-guided drainage; I&D = incision and drainage; RCT = randomised controlled trial; NA = not available

**Table 1/2.** Number of lesions at each location in each study

Author	Location of lesion (USD:I&D, *n*)									
	Submandibular	Parapharyngeal	Buccal	Pterygomandibular	Masseteric	Submental	Neck level 2	Parotid	Retropharyngeal space	Submandibular/paraphyaryngeal space
Mallick *et al*. (2023)	0	0	0	0	0	0	0	12:18 (30)	0	0
Biron *et al*. (2013)	7:8 (15)	1:1 (2)	0	0	0	0	0	0	0	0
Hassan and Gaafar (2021)	NA	NA	NA	NA	NA	NA	NA	NA	NA	NA
Fan and Tao (2021)	25.6%:41.2%	34.9%:23.5%	0	0	16.3%:11.8%	0	0	0	11.6%:23.5%	11.6% (USD only)
Dabirmoghaddam *et al*. (2017)	13:13 (26)	0:2 (2)	3:5 (8)	3:3 (6)	3:2 (5)	3:1 (4)	0:1 (1)	5:3 (8)	0:0 (0)	NA
Limardo *et al*. (2022)	Not enough information. Instead, the authors only reported the most common site overall: ‘the left submandibular space (40%), followed by the right submandibular (25%), submental, masticatory space (temporal, interpterygoid, submaseterine, …) and maxillary space.’									
Strassen *et al*. (2022)	0	0	0	0	0	0	Right parotid, 7:21 Left parotid, 11:17 Bilateral, 0:2	0	0	0

USD = ultrasonography-guided drainage; I&D = incision and drainage; NA = not available

### Primary outcomes

#### Recurrence

**Figure 2.**
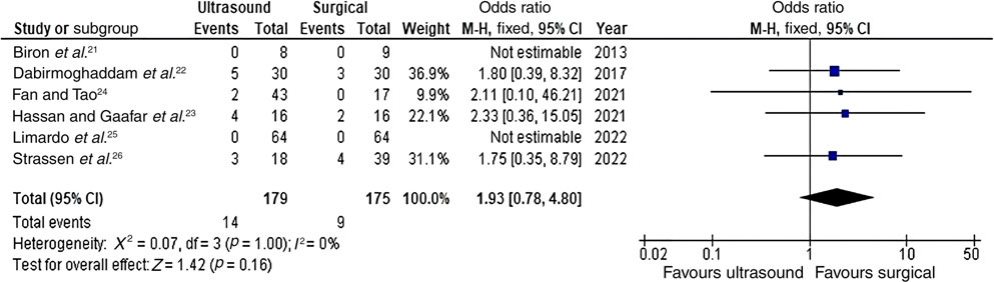
Forest plot for the odds ratio of ultrasound-guided drainage versus surgical drainage for deep neck space abscesses – recurrence. There was no statistically significant difference in the odds of recurrence between both groups. M-H = Mantel-Haenszel; CI = confidence interval; df = degrees of freedom

Figure 2 presents the meta-analysis findings for recurrence rate based on data from 6 studies involving 297 participants. No statistically significant difference was observed in the odds ratio for the recurrence rate between the 2 groups (odds ratio = 2.02, CI = 0.67 to 6.08, *p* = 0.21). A low level of heterogeneity was demonstrated among the studies (*I*^2^ = 0 per cent, *p* = 0.16).

#### Length of hospital stay

**Figure 3.**
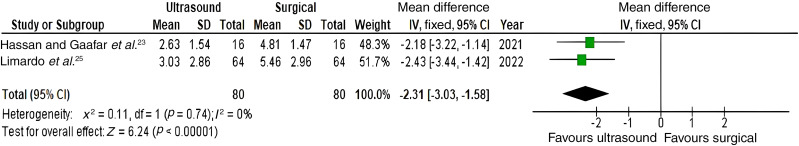
Forest plot for the mean difference in ultrasound-guided drainage versus surgical drainage for deep neck space abscesses – length of hospital stay (in days). The results indicate a statistically significant reduction in hospital stay duration in the ultrasonography-guided drainage group. SD = standard deviation; IV = inverse variance; CI = confidence interval; df = degrees of freedom

The length of hospital stays in 2 studies involving 160 participants is quantitatively depicted in the forest plot shown in Figure 3. A statistically significant difference in the mean length of hospital stay in days was observed, favouring the ultrasonography-guided drainage group over the incision and drainage group (means difference = −2.31, CI = −3.03 to −1.58, *p* < 0.00001). A low level of heterogeneity was demonstrated among the studies (*I*^2^ = 0 per cent, *p =* 0.74). Five additional studies, as reported by Biron *et al*.,^21^ Dabirmoghaddam *et al*.,^22^ Fan and Tao,^24^ Strassen *et al*.^26^ and Mallick *et al*.,^27^ also demonstrated the mean shorter length of hospital stay in days for the ultrasonography-guided drainage group (3.1 *vs* 5.2, *p* = 0.042; 5.47 *vs* 9.70, *p* < 0.001; 8 *vs* 10.8, *p* = 0.00028; 5.88 *vs* 7.33, *p* = 0.30; 5.416 *vs* 7.77, *p* = 0.03, respectively).

### Secondary outcomes

#### Complications

Fan and Tao reported one case of post-operative pneumonia in the surgery group, whereas the ultrasonography-guided drainage cohort had no complications.^24^ Limardo *et al*. reported two cases of persistent fever and increased oedema, one in each group. Both cases required re-operation with cervicotomy and wide drainage.^25^

Strassen *et al*. reported one complication of post-operative bleeding in the incision and drainage cohort. In contrast, there was no incidence of bleeding in the ultrasonography-guided drainage cohort despite repeated needle aspirations over multiple days.^26^ An incident of no pus punctured was seen in the ultrasonography-guided drainage cohort. Four cases of abscess recurrences were reported in the incision and drainage cohort and only three in the ultrasonography-guided drainage cohort. Two patients, one from each cohort, underwent surgical parotidectomy because of several recurrences.

In Mallick and colleagues’ study, one patient from the incision and drainage group had a bleeding problem, whereas in the ultrasonography-guided drainage group no patients reported bleeding despite repeated needle aspirations.^27^ Moreover, the authors observed a higher frequency of pain, swelling, localised heat and redness in the incision and drainage cohort.

#### Cosmetic appearance and/or scar formation

According to Hassan and Gaafar, ultrasonography-guided drainage resulted in significantly less scar formation than in those who underwent surgery (*p* = <0.001).^23^ Limardo *et al*. used a patient-scored scar assessment scale, which concluded that 98 per cent satisfaction was achieved with ultrasonography-guided drainage compared with 62 per cent satisfaction reported after incision and drainage.^25^

#### Cost savings

According to Biron *et al*., ultrasonography-guided drainage was associated with 41 per cent cost reductions compared with incision and drainage. This resulted in an estimated $8505.00 reduction in hospital bed costs per patient.^21^

### Methodological quality and risk of bias assessment

**Figure 4.**
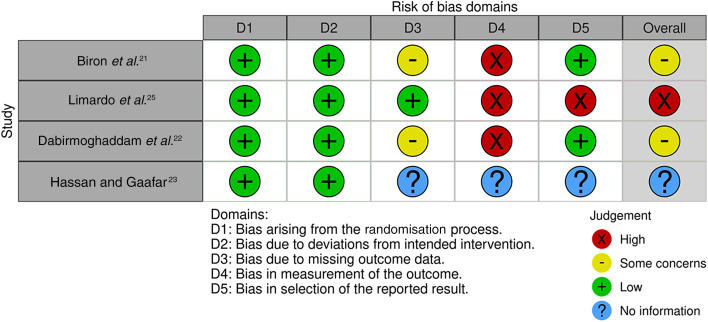
Visualisation tool showing assessment of the risk of bias using the Cochrane Collaboration Tool (ROB2) for randomised controlled trials.

**Figure 5.**
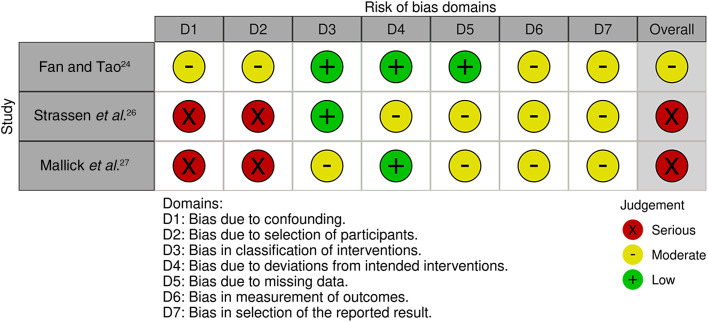
Visualisation tool showing the risk of bias assessment using the Cochrane Collaboration Tool for observational studies.

Four RCTs were assessed using the Risk Of Bias In Non-randomised Studies – of Interventions assessment tool, as seen in Figure 4. The results generally show that the studies had some bias concerns, and Limardo *et al*.'s study indicated a high risk of bias.^25^ Three observational studies were assessed using the Risk Of Bias In Non-randomised Studies – of Interventions tool, as seen in Figure 5.

## Discussion

This meta-analysis demonstrates that both ultrasonography-guided drainage, and incision and drainage can be used effectively in treating deep neck space abscesses, with no significant difference in the recurrence rate (*p* = 0.16). However, statistically significant shorter lengths of hospital stay were associated with ultrasonography-guided drainage compared to incision and drainage (*p* < 0.00001). All studies exhibited low heterogeneity (*I*^2^ = 0 per cent) and were therefore analysed using a fixed-effects model. Secondary outcome measures revealed that ultrasonography-guided drainage was associated with fewer surgical scars and post-operative bleeding events, and appeared to be more cost-effective.

The current study findings agreed with the previously published literature. Baatenburg de Jong and colleagues published one of the earliest case series supporting the use of ultrasonography-guided drainage as a cheap and effective alternative after successfully treating 5 patients with a parapharyngeal and/or retropharyngeal abscess without any complications or recurrences during 18–27 months of follow up.^28^ Notably, the length of hospitalisation ranged between two and three weeks, but that could have been related to the poor health of the recruited patients and little experience with the procedure; the drain was left in situ for several days after discharge had stopped.

Yeow *et al*. demonstrated the successful drainage of a deep retropharyngeal abscess and uniloculated parotid abscesses under ultrasound guidance; no incisions or only a small (5 mm or smaller) incision was needed to drain the pus, leading to better cosmetic outcomes and reducing pus contamination of the surrounding neck visceral spaces.^29,30^ The same researchers reported their experience in a trial that included 15 patients with uniloculated deep neck space abscesses, achieving an 87 per cent (13 out of 15) success rate without complications or recurrences during the 6 months of follow up.^31^ Two patients required incision and drainage due to the abscess progression and a diffuse spreading inflammatory process. Interestingly, the authors noted a shorter mean length of hospital stay (9 days) associated with the use of a catheter despite draining larger abscesses compared with using a needle (12 days). This suggests that small-calibre (7–8 French) pigtail catheters may be effective in treating uniloculated abscesses with liquefied pus content.

In a different case series, Al-Belasy demonstrated the resolution of masseteric space abscesses in 8 of the 11 patients (73 per cent).^10^ The failure in these cases was associated with higher average abscess volume (8.5 *vs* 17.5 ml). However, Brion and colleagues successfully drained a higher mean abscess volume (21 ml) in 8 patients using ultrasonography-guided drainage without recurrences.^21^

More recently, Wang and colleagues successfully drained a huge retropharyngeal abscess of 350 ml of tawny viscous pus using ultrasonography-guided drainage under local anaesthetic in a patient with pneumonia and suspected coronavirus disease 2019.^32^ In this case, ultrasonography-guided drainage had the added benefits of avoiding general anaesthesia risk for pneumonia patients and minimising infection spread via respiratory secretions and aerosols. Finally, Gudi and colleagues successfully treated 10 patients with submasseteric space abscesses using ultrasonography-guided drainage, and only 1 patient underwent incision and drainage because of infection spread.^33^

When thick pus or a narrowed puncture port and lumen create poor drainage, ultrasonography-guided drainage might be difficult to accomplish. However, abscess development is a dynamic process, and its viscosity depends on the timing of the drainage; it is easier to drain when abscess formation is completed and more difficult if the pus is viscous as a result of incompletely liquefied tissue.^24^ Nevertheless, a study by Lin *et al*. demonstrated successful drainage of thick pus in 14 patients with head and neck abscesses after implementing a contra-drainage method using multi-catheter and ultrasound guidance.^34^

Otolaryngologists might find it more challenging to drain multiloculated deep neck space abscesses because it is hard to open all septations effectively. Despite that, a poster of an RCT including 32 patients concluded that ultrasonography-guided drainage is a safe and effective alternative to incision and drainage for ‘uni- or multiocular deep neck abscesses’.^23^

The reported outcomes should be viewed considering this meta-analysis’ limitations. First, the number of studies included in the analysis was relatively small, with only 7 studies comprising 384 patients. This may not be sufficient to compare the two techniques accurately. Only one study reported a breakdown of costs associated with ultrasonography-guided drainage versus incision and drainage, and different healthcare systems account for different variables, therefore it is hard to come to a firm conclusion about cost savings. Two included studies were non-randomised, which introduces selection bias and affects the reliability of the results, raising the chances of type II error. Moreover, some of the included studies had moderate to high risks of bias, which lowers the quality of the meta-analysed data.

- Deep neck space abscesses are relatively common otolaryngology – head and neck surgery emergencies and are traditionally treated with surgical incision and drainage
- Patients undergoing incision and drainage often require general anaesthesia and may need airway stabilisation via tracheostomy. In addition, this modality is associated with a risk of neurovascular injury and can result in a cosmetically undesirable scar
- Ultrasonography-guided drainage is a minimally invasive and inexpensive tool that could overcome the abovementioned drawbacks
- It was found ultrasonography-guided drainage is associated with a shorter hospital stay and appeared to be more cost-effective, with better cosmetic outcomes
- There was no statistically significant difference in the recurrence rate between ultrasonography-guided drainage, and incision and drainage
- Further well-designed multicentre prospective studies with standardised outcomes reporting are needed to increase confidence in the use of ultrasonography-guided drainage

A quantitative meta-analysis of secondary outcomes was not possible because of the limited data available on these outcomes. In addition, we excluded five relevant studies from the meta-analysis (Figure 3) because of inadequate data reporting, despite efforts to contact the corresponding authors by email. As the results of these studies favoured ultrasonography-guided drainage, they would have been unlikely to divert the direction of our results, but more precise effect estimates and corresponding CIs would have been obtained. Future studies should aim to standardise outcome reporting and ensure all data are included to strengthen the available evidence within the literature.

## Conclusion

This is the first systematic review and meta-analysis with robust methodology to compare ultrasonography-guided drainage with incision and drainage in adult patients with deep neck space abscesses. It is crucial for head and neck surgeons to consider using ultrasonography-guided drainage as a safe and effective alternative to incision and drainage, especially when deep neck space abscesses are uninoculated and well-defined, and when general anaesthesia is undesirable. There may be cost savings associated with the reduction in hospital stay and better cosmetic outcomes, but these are not the primary outcomes of this study.

The authors suggest further RCTs that adhere to the Consolidated Standards of Reporting Trials guidelines^35^ to increase confidence in the use of ultrasonography-guided drainage and provide a stronger evidence base to support its usage. There is clear heterogenous outcomes reporting amongst the published studies, therefore developing core outcomes sets using consensus methods is implicated to reduce risk of bias and foster methodological research in deep neck space abscesses.

## Data Availability

The datasets generated and analysed during the current study are available from the corresponding author on reasonable request.
